# Eco-Friendly Hybrid PLLA/Chitosan/*Trichoderma asperellum* Nanomaterials as Biocontrol Dressings against Esca Disease in Grapevines

**DOI:** 10.3390/polym14122356

**Published:** 2022-06-10

**Authors:** Mariya Spasova, Nevena Manolova, Iliya Rashkov, Mladen Naydenov

**Affiliations:** 1 Laboratory of Bioactive Polymers, Institute of Polymers, Bulgarian Academy of Sciences, Acad. G. Bonchev St, bl. 103A, BG-1113 Sofia, Bulgaria; manolova@polymer.bas.bg (N.M.); rashkov@polymer.bas.bg (I.R.); 2 Department of Microbiology, Agricultural University, BG-4000 Plovdiv, Bulgaria; mladen@au-plovdiv.bg

**Keywords:** PLLA, electrospinning, chitosan, *Trichoderma asperellum*, *Phaeomoniella chlamydospora*, *Phaeoacremonium aleophilum*, esca

## Abstract

Fungi constitute the largest number of plant pathogens and are responsible for a range of serious plant diseases. *Phaeomoniella chlamydospora* (*P. chlamydospora*) and *Phaeoacremonium aleophilum* (*P. aleophilum*) are the main fungal pathogens causing esca disease in grapevines. On the other hand, there are beneficial microorganisms such as *Trichoderma* spp., which are able to control the growth of many phytopathogens. In the present study, innovative, eco-friendly hybrid nanomaterials were created by electrospinning PLLA, followed by the formation of a film of chitosan/*Trichoderma asperellum (T. asperellum)* spores on the fibers. The polymer carrier used in this study plays an active role in ensuring the viability of the biological agent during storage and, when placed in contact with moisture, ensures the agent’s normal development. Oligochitosan, as well as low molecular weight and high molecular weight chitosan, were used. The effects of chitosan molecular weight on the dynamic viscosity of chitosan solutions, film formation, mechanical properties, spore incorporation and growth were studied. The morphology of the prepared nanomaterials, and the presence of a film based on the formation of chitosan/*T. asperellum* spores on the PLLA fibers, were examined using scanning electron microscopy (SEM). The surface chemical compositions of the fibrous materials were studied using attenuated total reflection Fourier transform infrared spectroscopy (ATR-FTIR). The mechanical properties of the obtained materials were also tested. The microbiological screening that was performed revealed that the eco-friendly hybrid nanomaterials incorporated with the beneficial microorganism, *T. asperellum*, to hamper the growth of the pathogenic *P. chlamydospora* and *P. aleophilum* fungi. The suppression rate depended on the viscosity of the chitosan solution used for the film formation. The use of oligochitosan resulted in the most effective infection of the material with *T. asperellum* spores. The environmentally friendly hybrid nanomaterials obtained in this study—in which the bioagent was embedded—are promising bioactive dressings for protecting grapevines against esca disease.

## 1. Introduction

In recent years, plant diseases caused by bacterial, fungal and viral infections have resulted in significant reductions of crop yields and in significant economic and social issues [1]. Esca is a grapevine trunk disease that occurs within vineyards worldwide [2]. Recently, esca has been increasing in incidence, causing extensive damage, great financial losses and reducing the quality of both grapes and the wines made from the affected grapes. Many pathogens (mainly fungi) are involved, such as *Phaeomoniella chlamydosporum*, *Phaeoacremonium aleophilum*, *Botryosphaeria* spp., *Cylindrocarpon* spp., *Eutypa lata* and *Phomopsis viticolaand* [3]. It is already known that vine wood becomes infected after pruning, with fungi developing and progressively colonizing the tissues of the grapevine. The disease symptoms include the appearance of dark red or yellow stripes on the grapevine leaves, which eventually dry and become necrotic [4]. The disease has two forms: a chronic form, which lasts for several years and for which the symptoms are not visible every year; and an acute form, which results in the death of the entire plant within a few days [5].

Until 2003, the prevention and control of the disease was achieved through the use of sodium arsenite fungicides. However, sodium arsenite was banned in France in 2001 and by the rest of Europe in 2003, due to it being highly toxic and carcinogenic [6]. Nowadays, the most effective approach against esca is to prevent infection. Disinfection of a nursery is normally conducted using hot water; however, some grapevine varieties are very sensitive to this method. Another method is by removing and burning branches and dead vines and pruning wastes. Other preventive approaches need to be developed.

The electrospinning technique was developed intensively during the last decade for the fabrication of continuous fibers, with diameters ranging from 20 nm to several microns [7]. Polymer, metal, ceramic and composite nanofibers became easily able to be fabricated [8]. Electrospun nanofibers possess high surface-to-volume ratios and high porosity [9], making them suitable candidates for use in a variety of applications, such as tissue engineering [10], wound dressing [11], drug delivery [12], filtration [13], composite reinforcement [14], protective clothing [15], smart textiles [16], batteries [17], capacitors [18], sensors [19], catalysts [20], etc.; however, the use of electrospun nanofibers in agriculture is relatively new [21].

Until now, there have been scarce data in the literature discussing the use of the electrospun fibrous materials in agriculture—a sector in which the protection of vineyards has become an emerging field of interest. Micro- and nanofibrous materials prepared by electrospinning could become modern plant protection products, possessing the desired biological effects to fight against phytopathogenic fungal infections, but without the harmful impacts and side effects. However, few research studies have demonstrated the fabrication and potential application of using fibrous materials to protect grapevines against esca disease. The first report investigated the electrospinning of soy protein/polyvinyl alcohol and soy protein/polycaprolactone onto rayon membranes. It was predicted that the fabricated hybrid material should prevent the penetration of fungal spores [22]; however, some spores penetrated the membranes. The authors concluded that for future work, antifungal agents should be loaded into the fibers. Another research group proposed the use of poly(D,L-lactide-*co*-glycolide) and poly(butyleneadipate-*co*-terephthalate) for application in vineyards. Polyhexamethylene guanidine, an antimicrobial polymer, was incorporated into the nonwovens as well. The authors concluded that spore penetration could not be avoided due to the diverse range of pore-size distributions in all electrospun fibrous membranes. However, an efficient and breathable barrier membrane that prevented spore penetration was produced using an antifungal blend for electrospinning [23].

The results and the conclusions from the two presented studies were a basis for us to develop our innovative plant-protecting fibrous materials, designed to protect against penetration by the major pathogens that cause esca disease in grapevines. In our previous study, we created nanosized materials based on biodegradable and biocompatible polymers and metal oxide nanoparticles. Composite fibers, based on poly(3-hydroxybutyrate), nanosized TiO_2_-anatase and chitosan oligomers (COS), with antifungal activity were fabricated using a combination of electrospinning and electrospraying techniques [24]. Moreover, the incorporation of ZnO nanoparticles into cellulose acetate nanofibers led to the preparation of fibrous membranes with water-repellent and antifungal properties [25].

The other approach we used was the incorporation of chemical compounds with strong antimicrobial and antifungal activity into the fibrous materials. For instance, 8-hydroxyquinoline and its derivatives belong to a group of chemical fungicides and possess antimicrobial [26,27], antiviral [28] and antifungal properties [29,30].

In our previous studies, we prepared fibrous materials based on biodegradable and biocompatible polymers loaded with an antifungal agent for active protection against spore penetration and plant infection in vineyards [31,32,33]. The incorporation of diverse 8-hydroxyquinoline derivatives into the fibers imparted to them considerable antifungal activity that acted against *P. chlamydospora* and *P. aleophilum*. The novel fibrous materials obtained, which were loaded with the chemical fungicide, could be potential candidates for application in agriculture to protect grapevines against esca-associated fungi.

Modern agriculture demands the development of effective and ecologically safe devices in order to overcome the widely spread use of synthetic pesticides. For instance, there are a considerable number of beneficial microorganisms that are good biocontrol agents of pathogenic fungi. It is well known that *Penicillum*, *Trichoderma*, *Bacillus* and *Streptomyces* fungi and bacteria produce enzymes that are able to degrade natural polymers such as chitin and its derivatives, which are the construction elements of the cell walls of certain plant pathogenic fungi [34,35,36,37]. Using such biocontrol agents can result in crop increases and diminish the pollution of the environment with pesticides.

In the present study, an innovative approach is proposed, combining the useful and valuable properties of biopolymers with those of the bioagent in order to create eco-safe materials for application in agriculture. The beneficial microorganism, *T. asperellum*, is used as the bioagent. The results show that PLLA fibers coated with chitosan possess excellent mechanical properties and play an active role in promoting the viability of the biological agent during storage and, when placed in contact with moisture, ensure the agent’s normal development. The obtained results show that the *T. asperellum* spores incorporated into the polymer matrix exhibit antagonistic activity against *P. chlamydospora* and *P. aleophilum* fungal species. Thus, the novel agropharmaceutical devices created in this study could be used to control certain diseases, such as esca, on grapevines.

## 2. Materials and Methods

### 2.1. Materials

Poly(L-lactide) (PLA)(Ingeo™ Biopolymer 4032D, NatureWorks LLC—Minnetonka, MN, USA; M_W_ = 259,000 g/mol, M_W_/M_n_ = 1.94, as determined by size-exclusion chromatography using polystyrene standards), chitosan oligomer (COS)(Kitto Life Co. LTD, Pyeongtaek-si, Gyeonggi-do, Korea; average molecular weight 3000–5000 g/mol), low molecular weight chitosan (LMW) (50,000–190,000 Da, 20–300 cP (1% *w*/*w* CH_3_COOH 1%), degree of deacetylation 75–85%; Sigma–Aldrich, St. Louis, MO, USA) and high molecular weight chitosan (HMW)(Mr~600,000 g/mol, degree of deacetylation 80%; Sigma–Aldrich, St. Louis, MO, USA) were used in the study. Dichloromethane, ethanol and glacial acetic acid (Merck; Darmstadt, Germany) were of an analytical grade of purity and were used as received. Potato dextrose agar medium was delivered by Merck (Darmstadt, Germany).

The *T. asperellum* microorganism was obtained from the collection at the Department of Microbiology, Agricultural University, Plovdiv, Bulgaria.

The fungi *P. chlamydospora* CBS 239.74 and *P. aleophilum* CBS 631.94 were purchased from Westerdijk Fungal Biodiversity Institute (Utrecht, The Netherlands).

### 2.2. Preparation of Fibrous PLLA Materials by Electrospinning

The PLLA fibrous material was prepared by electrospinning its solution with a concentration of 10 wt% in DCM/EtOH (DCM/EtOH = 90/10). The spinning solution was placed in a syringe (5 mL) equipped with a metal needle (gauge: 20GX1½″), which was connected to the positively charged electrode of a DC high-voltage power supply. The electrospinning was conducted at a constant applied voltage (25 kV), with a constant distance between the conical nozzle and the collector (15 cm) and at a delivery rate of 3 ml h^−1^ for the PLA solution (pump Syringe Pump NE-300; New Era Pump Systems, Inc., (Farmingdale, NY, USA). The rotating rate of the collector was 1000 rpm at a temperature of 25 °C and humidity of 50%. After the electrospinning, the samples were placed under a vacuum for 72 h to remove any solvent residue.

### 2.3. Coating of Fibrous PLLA Materials with Chitosan or Chitosan/T. asperellum Spores

Chitosan oligomer was dissolved in distilled water at concentration of 0.5 wt%. Additionally, for the dissolution of the LMW and HMW chitosans (concentration—0.5%), glacial acetic acid was added, drop by drop, until a pH of 5 was reached.

*T. asperellum* was cultured in a liquid medium containing glucose (25 g/L) and corn steep liquor (25 mL/L), in a bioreactor for 5 days at 28 °C. The culture liquid was enriched with fungal conidia cultivated on solid culture media for 7 days at 28 °C. Fungal spores were obtained by centrifugation (4000 rpm for 30 min). The concentration of fungal spores was 1 × 10^8^/mL. An amount of 1 ml of the fungal suspension was added to 25 mL of the preliminary prepared chitosan solution.

Then, the PLLA fibrous mats were immersed into the chitosan solutions or chitosan/spore suspensions. The mats were left in the solutions/suspensions for 30 min, then withdrawn from the solutions, blotted carefully and dried to a constant weight.

### 2.4. Characterization

The dynamic viscosities of the solutions were measured using a Brookfield LVT viscometer ((Middleboro, MA, USA) equipped with an adaptor for small samples, a spindle and a camera (SC 4-18/13 R) at 20 ± 0.1 °C. The spinning solutions were measured in triplicate and the mean values with their standard deviations were calculated.

The morphology of the prepared materials was evaluated by scanning electron microscopy (SEM). The samples were vacuum-coated with gold prior to observation by SEM (Jeol JSM-5510, Tokyo, Japan). The mean fiber diameter and the spore sizes were estimated using ImageJ software [38].

IRAffinity-1 spectrophotometer (Shimadzu Co., Kyoto, Japan) equipped with a MIRacle™ATR (diamond crystal, depth of penetration of the IR beam into the sample—about 2 μm) accessory (PIKE Technologies, Fitchburg, WI, USA) was used to record attenuated total reflection Fourier transform infrared (ATR-FTIR) spectra. The spectra were recorded in the range of 600–4000 cm^−1^ with a resolution of 4 cm^−1^. All spectra were corrected for H_2_O and CO_2_ using an IRsolution software program.

Easy Drop (DSA20E Krűss GmbH) drop shape analysis system (Hamburg, Germany) was used to determine the values of the static contact angles. A sessile drop of deionized water with a volume of 10 μL, controlled by a computer dosing system, was deposited onto the material’s surface. The collected data were the averages of 10 measurements for each sample.

The tensile characteristics of the samples were evaluated using a single-column system for mechanical testing (INSTRON 3344), equipped with a loading cell of 50 N and Bluehill universal software. The strain rate was 10 mm/min, the initial length between the clamps was 40 mm and the room temperature was 21 °C. The width, the length and the thickness of the tested specimens were 20 mm, 60 mm and 400 μm, respectively. The samples were cut in the collector rotation direction. For the sake of statistical significance, 10 specimens of each sample were tested, after which the average values of Young’s modulus, the ultimate stress and maximum deformation at break were determined.

### 2.5. Microbiological Tests

*P. chlamydospora* CBS 239.74 and *P. aleophilum* CBS 631.94 were purchased from Westerdijk Fungal Biodiversity Institute (Utrecht, The Netherlands). The interaction between the fungal antagonist (*T. asperellum*) incorporated into the fibrous biomaterials and the pathogenic fungi (*P. chlamydospora* and *P. aleophilum*) was determined by in vitro studies carried out using potato dextrose agar medium. The surface of the solid agar was inoculated with a suspension of pathogenic fungi culture with a fungi concentration of 1 × 10^5^ colony-forming units (CFU)/mL and on the surface of the agar in each Petri dish, one electrospun biomaterial (17 mm in diameter) was placed. The Petri dishes were incubated for 96 h at 28 °C and, subsequently, the growth of the colonies of the phytopathogenic fungus and the antagonist fungus was measured.

### 2.6. Statistical Analysis

The data were displayed as means ± standard deviation (SD).

## 3. Results and Discussion

### 3.1. Morphological Analysis

In our previous studies, we demonstrated the successful preparation of electrospun biohybrid materials for plant biocontrol. This was achieved by incorporating *T. viride* spores into chitosan/polyethylene oxide and chitosan/polyacrylamide fibers. It was shown that the electrospun biohybrid materials inhibited the growth of diverse phytopathogenic strains (*Fusarium, Alternaria*) when placed at suitable growth conditions. Moreover, coating the plant sprouts with the biohybrids was easily achieved through direct electrospinning [39]. However, the mechanical properties of the obtained fibrous mats needed to be improved. Therefore, for vineyard protection against the pathogens causing esca disease, we decided to develop a novel strategy for the preparation of biomaterials based on electrospun PLLA mats (which possessed good mechanical properties) followed by the formation of a thin biofilm of chitosan/*T. asperellum* spores. The chitosan was selected for its inherent antibacterial properties and for the fact that it can elicit plant defense reactions. Thus, the combination of PLLA (which has good physico-mechanical properties) and the biological activity of chitosan (which has beneficial microorganisms) can lead to the preparation of novel biohybrid materials which can prevent microorganism invasions.

**Figure 1:**
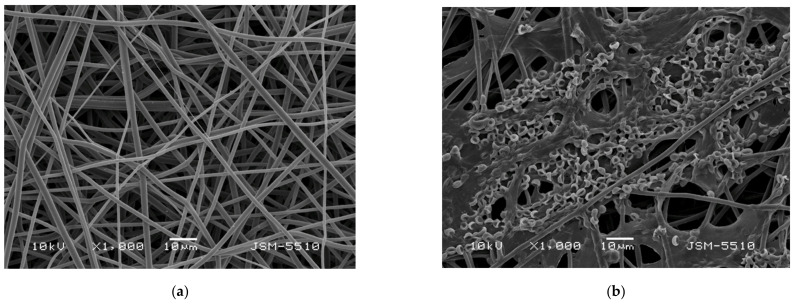

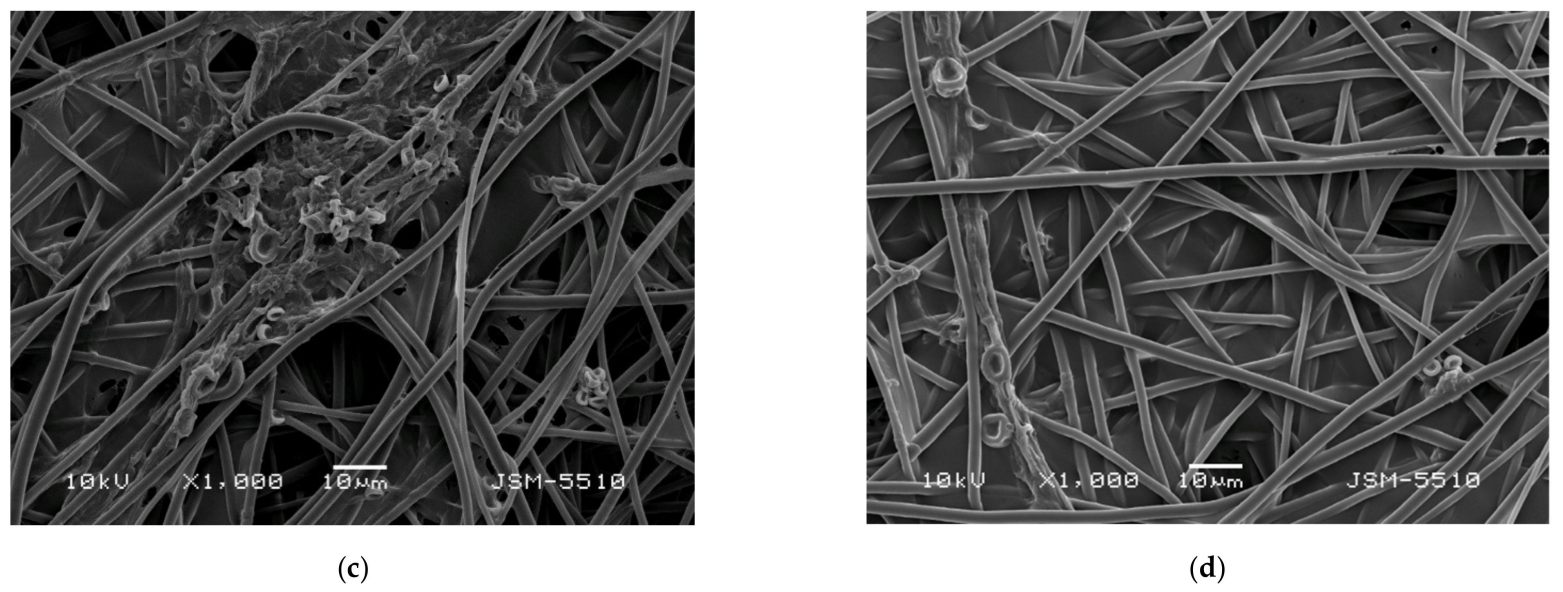
Representative SEM images of (**a**) a PLLA mat, (**b**) a PLLA mat coated with oligochitosan/*T. asperellum* spores, (**c**) a PLLA mat coated with LMW chitosan/*T. asperellum* spores and (**d**) a PLLA mat coated with HMW chitosan/*T. asperellum* spores; magnification ×1000.

SEM images of the obtained PLLA and the PLLA mat coated with a film of chitosan, with different molecular weights and *T.* *asperellum* spores, are presented in Figure 1. The electrospinning of PLLA solutions with a concentration of 10 wt% resulted in the preparation of fibers with an average fiber diameter of 1260 ± 192 nm (Figure 1a). The measured values for the PLLA fiber diameters were in a fairly good agreement with literature data [40]. As can be seen from Figure 1a, the process conditions and solution properties used resulted in the fabrication of defect-free cylindrical fibers without pores along the fiber lengths.

In order to investigate the film formation ability and the effect of the chitosan molecular weight on the solution viscosity, on the material’s wettability and on the mechanical properties, three types of chitosans were used: oligochitosan, LMW chitosan and HMW chitosan. The dynamic viscosities of the prepared solutions of oligochitosan, LMW chitosan and HMW chitosan were 35 cP, 99 cP and 308 cP, respectively.

The morphology of the PLLA fibers coated with chitosan/*T. asperellum* spores are shown in Figure 1b–d. The coating of the PLLA fibers with chitosan increased the fiber diameters to 1388 nm ± 220 nm (for the PLLA mat coated with HMW chitosan). Furthermore, it can be seen that the highest number of spores and the most uniform spore distribution on the PLLA fibers was achieved when oligochitosan was used (Figure 1b). The number of spores determined from the SEM micrographs was about 150 spores per 13,000 μm^2^ for the PLLA mat coated with oligochitosan/*T. asperellum*, ~60 spores/13,000 μm^2^ for PLLA mat coated with LMW chitosan/*T. asperellum* and ~20 spores for the same area for the PLLA mat coated with HMW chitosan/*T. asperellum* spores. This could be explained by the low oligochitosan solution viscosity value (35 cP) and the ability of the solution to penetrate more easily into the fibrous PLLA mat. As seen from the SEM micrograph (Figure 1b), the surface of the PLLA mat coated with oligochitosan was saturated with many spores. Increasing the molecular weight of the chitosan resulted in an increase of the solution viscosity, thus impeding the infection of the mat with spores. Nevertheless, it is easily visible that *T. asperellum* spores presented in all PLLA fibrous materials coated with chitosan films.

### 3.2. Contact Angle Measurements

**Figure 2:**
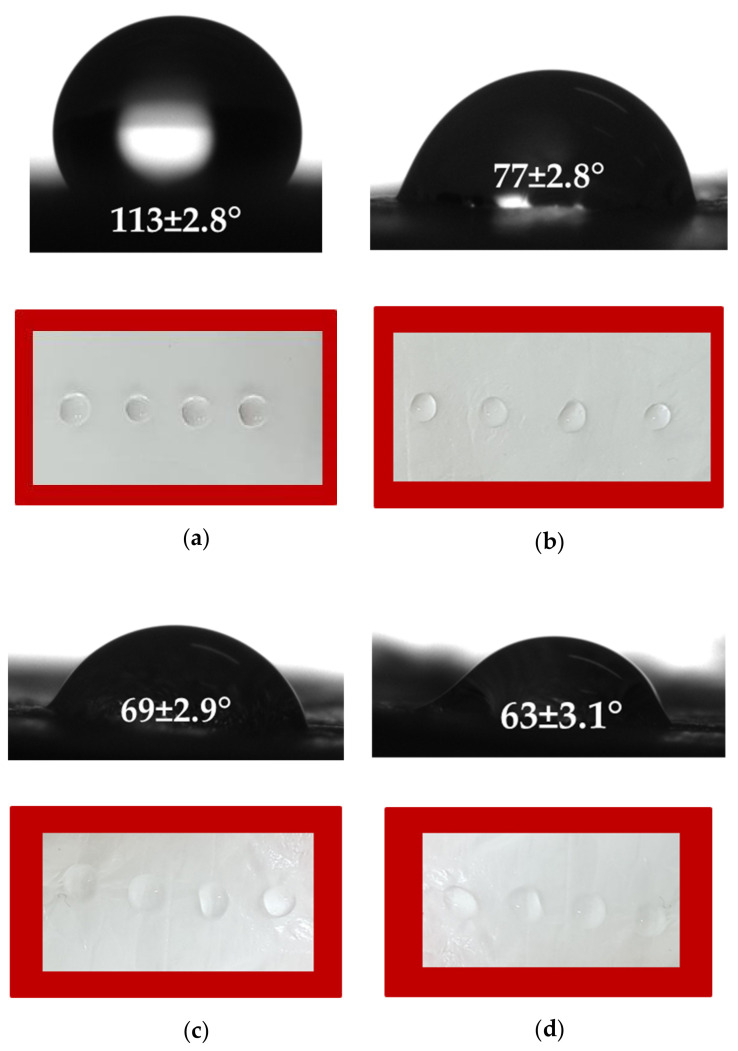
Digital images of water droplets spotted on the surface of fibrous biomaterials: (**a**) PLLA mat, (**b**) PLLA mat coated with oligochitosan, (**c**) PLLA mat coated with LMW chitosan and (**d**) PLLA mat coated with HMW chitosan.

Generally, chitosan oligomers are water-soluble. Chitosans with a low degree of deacetylation are insoluble in aqueous solutions at pH > 6. However, they can be easily dissolved in acidic aqueous solutions below a pH of 6. [41]. Surface colonization by fungi relies on many factors. However, the initial stage of interaction of fungi with a host surface depends on non-specific interactions involving hydrophobicity, charge, or other surface properties. Therefore, the ability of the material’s surface to be wetted is a key factor on which the adhesion of pathogens depends. Thus, it is of crucial importance to measure the wettability of the surface of fibrous material obtained in the present study that will be in contact with fungal spores. Digital images of the water droplets deposited on the surface of the fibrous biomaterials and the measured values of the water contact angles are presented in Figure 2. The fibrous mat based on PLLA had a water contact angle of ~113°, possessing a hydrophobic surface (Figure 2a). The coating of PLLA mat with oligochitosan resulted in a decrease of the water contact angle to ~77° (Figure 2b). The measured values of the contact angles of the PLLA coated with LMW and HMW chitosans were ~69° and ~63°, respectively. The decrease of the water contact angle values the increase the chitosan molecular weight is most likely due to higher values of dynamic viscosity—especially for the HMW chitosan solution—leading to the formation of a thicker chitosan film on the PLLA mat surface.

### 3.3. FTIR Spectroscopic Analysis

**Figure 3:**
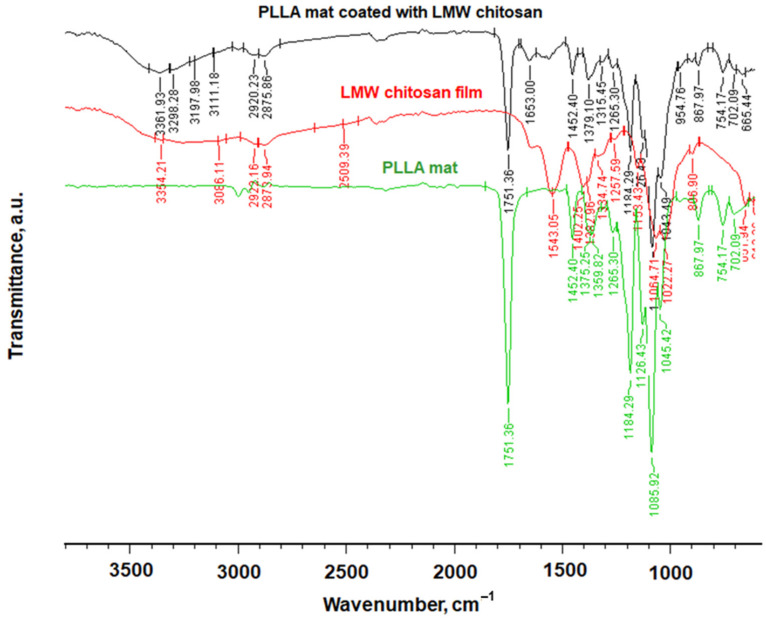
FTIR spectra of PLLA mat, LMW chitosan film and PLLA mat coated with LMW chitosan.

Fourier transform infrared spectroscopy (FTIR) was used for chemical characterization of the materials obtained in the present study. The recorded FTIR spectra of PLLA mat, LMW chitosan film and PLLA mat coated with LMW chitosan are shown in Figure 3. Characteristic bands for PLLA were detected at 1751 cm^−1^ for the C=O groups and at 1184 cm^−1^ for C-O-C groups. Characteristic stretching frequencies for C-O at 1085 cm^−1^ and bending frequencies for -CH_3_ asymmetric and -CH_3_ symmetric were observed at 1452 cm^−1^ and 1359 cm^−1^, respectively. In the spectrum of the chitosan film, a broad band in the range from 3600 cm^−1^ to 2750 cm^−1^ was detected, which was attributed to υ_NH_ and υ_OH_ vibrations and stretching vibrations of the -CH_2_-group at 2873 cm^−1^. The band at 1653 cm^−1^ corresponded to the amide I band and the band between 691 (cm^−1^) and 1560 (cm^−1^) corresponded to stretching C-C. C-O or C-N bonds were also detected.

In the spectrum of the PLLA mat coated with LMW chitosan, in addition to the characteristic bands of the PLLA, characteristic bands of the chitosan were also detected. This proved that a hybrid material composed of a PLLA mat and coated with chitosan film had been prepared.

### 3.4. Analysis of Physico-Mechanical Characteristics

**Figure 4:**
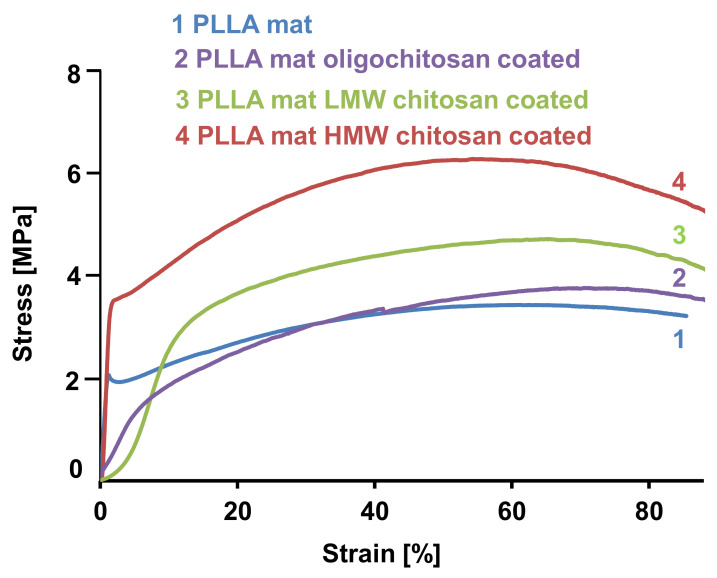
Stress–strain curves of: 1. A PLLA mat; 2. A PLLA mat coated with oligochitosan; 3. A PLLA mat coated with LMW chitosan; and 4. A PLLA mat coated with HMW chitosan.

Physico-mechanical properties of the fibrous samples prepared in the present study were measured using a single-column testing machine. The stress–strain curves obtained for the PLLA mat, the PLLA mat coated with oligochitosan, the LMW chitosan and the HMW chitosan are shown in Figure 4. The fibrous mat composed of neat PLLA showed a tensile strength of 2.8 MPa. This value is in good agreement with the values found in the literature [42]. The coating of the PLLA fibers with chitosan film resulted in enhancements of the mechanical properties of the hybrid fibrous materials. The mechanical characteristics determined for the PLLA mat coated with oligochitosan were a little bit higher than those of the neat PLLA mat. The values of the tensile strength of the PLLA mats coated with LMW and HMW chitosan were ca. 4.2 and 6.1 MPa, respectively. This increase in mechanical behavior is most likely due to the inter-PLLA fiber bonding by the chitosan film.

### 3.5. Fungal Morphology and Fungi Suppression Ability

*P. aleophilum* and *P. chlamydospora* are the two main fungi responsible for the infection of grapevines with the esca disease. It is believed that the vines’ pruning wounds are the main “entrance” for penetration by the fungal spores and subsequent infection of the plants. On the other hand, *Trichoderma* spp. is an effective and eco-friendly biocontrol agent with a relatively low cost, which prevents or obstructs the development of pathogenic organisms.

**Figure 5:**
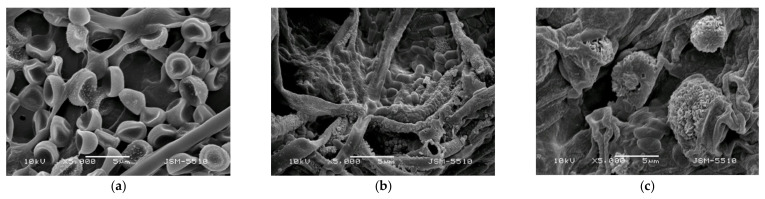
SEM images of the (**a**) *T. asperellum* spores, (**b**) *P. chlamydospora* conidia and (**c**) *P. aleophilum* conidia.

Initially, the morphology of the microorganisms used in the present study was observed by SEM analysis. The diameters and the lengths of the spores and conidia were measured using ImageJ software. The SEM images of the *T. asperellum* spores are shown in Figure 5a. The diameter of the spores was ~3 µm. The diameter and length of the *P. chlamydospora* conidia were ~0.75–1.2 µm and 1.8–2.3 µm, respectively (Figure 5b). The morphology of the *P. aleophilum* conidia is shown in Figure 5c and the measured diameter and length of the *P. aleophilum* conidia from the SEM micrograph were ~1.1–1.5 µm and 2.5–3.5 µm, respectively.

In our preliminary studies, we determined that chitosan, with different molecular weights and incorporated into the agar medium, did not inhibit the growth of the two pathogenic fungi. Thereafter, we performed microbiological tests to ascertain the ability of the created hybrid biomaterials containing the beneficial microorganism to suppress the development and growth of *P. chlamydospora* and *P. aleophilum*.

**Figure 6:**
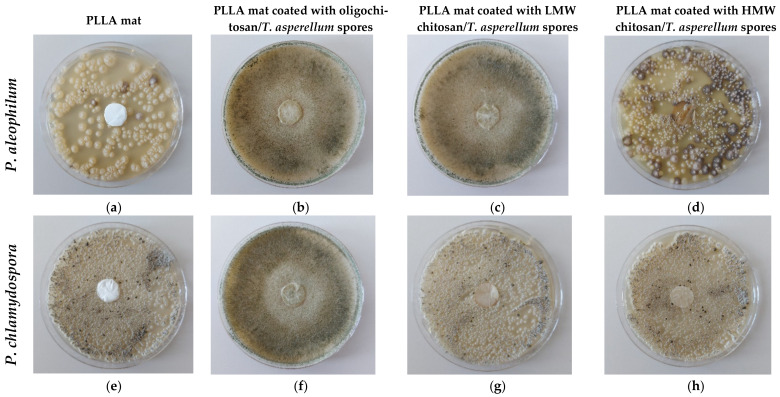
Digital images of the behavior test of the mats against *P. aleophilum* (**a**–**d**) and *P. chlamydospora* (**e**–**h**): (**a**,**e**) a PLLA mat; (**b**,**f**) a PLLA mat coated with oligochitosan/*T. asperellum* spores; (**c**,**g**) a PLLA mat coated with LMW chitosan/*T. asperellum* spores; and (**d**,**h**) a PLLA mat coated with HMW chitosan/*T. asperellum* spores.

The discs of the fibrous mats of the PLLA mat—the PLLA mat coated with oligochitosan/*T. asperellum*, coated with LMW chitosan/*T. asperellum* spores or coated with HMW chitosan/*T. asperellum* spores—with diameters of 17 mm, were placed in contact with *P. chlamydospora* and *P. aleophilum*. The ability of *T. asperellum* to hamper the growth of the pathogenic fungi incorporated into the hybrid materials is shown in Figure 6. The PLLA mat did not show any activity and the normal growth of *P. chlamydospora* and *P. aleophilum* is clearly visible (Figure 6a,e). As seen from Figure 6, the PLLA mat coated with oligochitosan/*T. asperellum* spores was the most effective in suppressing the two pathogenic fungi. The whole Petri dish surface was covered with the *Trichoderma* mycelium, completely hampering the growth of *P. aleophilum* and *P. chlamydospora.* The PLLA mat coated with LMW chitosan/*T. viride* spores was less effective in suppressing the pathogenic growth. Nevertheless, *Trichoderma* parasitized on *P. aleophilum*. However, against *P. chlamydospore*, the *Trichoderma* parasitism was almost absent. No parasitism of the incorporated *Trichoderma* was detected in the PLLA mat coated with HMW chitosan/*T. asperellum* spores (Figure 6d,h), which was placed in contact with *P. aleophilum* and *P. chlamydospora*. This was most likely due to an insufficient number of spores being incorporated into this material—which was proven by the SEM analysis.

The effect of chitosan for ensuring the viability of the biological agent was expressed and, when placed in contact with moisture, ensured the biological agent’s normal development. The PLLA ensured the good mechanical properties of the composite materials. The obtained results showed that the *Trichoderma* spores were alive and could suppress the growth of the pathogenic fungi when incorporated into chitosan films, which were formed from diluted solutions and assisted in uniform film formation and spore incorporation. The optimal manner for incorporating *Trichoderma* spores into the materials was through the use of a suspension based on oligochitosan and *T. asperellum* spores.

## 4. Conclusions

Novel hybrid biomaterials based on PLLA/chitosan/*T. asperellum* were successfully developed. The combination of polymers with the biocontrol agent by using the electrospinning and film formation techniques resulted in the preparation of eco-friendly nanomaterials with good mechanical properties (tensile strength 6.1 MPa), ensuring the viability of the *T. asperellum* spores. The effects of the chitosan molecular weight (oligochitosan, LMW chitosan and HMW chitosan) and viscosity on the physico-chemical and biological properties of the obtained materials were studied. The most effective suppression of the growth of the pathogenic *P. chlamydospora* and *P. aleophilum* fungi was achieved using oligochitosan for the incorporation of *T. asperellum* spores. Thus, the created eco-friendly hybrid nanomaterials possess the potential to be used as biocontrol dressings against esca disease in grapevines.

## Data Availability

Data is contained within the article.
